# Hyperspectral Canopy Sensing of Wheat Septoria Tritici Blotch Disease

**DOI:** 10.3389/fpls.2018.01195

**Published:** 2018-08-17

**Authors:** Kang Yu, Jonas Anderegg, Alexey Mikaberidze, Petteri Karisto, Fabio Mascher, Bruce A. McDonald, Achim Walter, Andreas Hund

**Affiliations:** ^1^Crop Science Group, Institute of Agricultural Sciences, ETH Zurich, Zurich, Switzerland; ^2^Plant Pathology Group, Institute of Integrative Biology, ETH Zurich, Zurich, Switzerland; ^3^Plant Breeding and Genetic Resources, Strategic Research Division Plant Breeding, Agroscope, Nyon, Switzerland

**Keywords:** field-based phenotyping, hyperspectral remote sensing, partial least squares discriminant analysis, plant phenotyping, plant spectroscopy, resistance breeding, Septoria tritici blotch, wheat disease

## Abstract

Producing quantitative and reliable measures of crop disease is essential for resistance breeding, but is challenging and time consuming using traditional phenotyping methods. Hyperspectral remote sensing has shown potential for the detection of plant diseases, but its utility for phenotyping large and diverse populations of plants under field conditions requires further evaluation. In this study, we collected canopy hyperspectral data from 335 wheat varieties using a spectroradiometer, and we investigated the use of canopy reflectance for detecting the Septoria tritici blotch (STB) disease and for quantifying the severity of infection. Canopy- and leaf-level infection metrics of STB based on traditional visual assessments and automated analyses of leaf images were used as ground truth data. Results showed (i) that canopy reflectance and the selected spectral indices show promise for quantifying STB infections, and (ii) that the normalized difference water index (NDWI) showed the best performance in detecting STB compared to other spectral indices. Moreover, partial least squares (PLS) regression models allowed for an improvement in the prediction of STB metrics. The PLS discriminant analysis (PLSDA) model calibrated based on the spectral data of four reference varieties was able to discriminate between the diseased and healthy canopies among the 335 varieties with an accuracy of 93% (Kappa = 0.60). Finally, the PLSDA model predictions allowed for the identification of wheat genotypes that are potentially more susceptible to STB, which was confirmed by the STB visual assessment. This study demonstrates the great potential of using canopy hyperspectral remote sensing to improve foliar disease assessment and to facilitate plant breeding for disease resistance.

## Introduction

Septoria tritici blotch (STB), caused by a fungal pathogen *Zymoseptoria tritici*, is one of the most damaging foliar diseases in wheat, causing crop yield losses and resulting in higher production costs due to fungicide applications (Fones and Gurr, 2015; Torriani et al., 2015). It will be difficult to meet the growing global demands for wheat whilst reducing the environmental costs associated with wheat production without better control of STB based on breeding varieties with improved STB resistance (O'Driscoll et al., 2014). Remote sensing and mapping techniques have been suggested as new tools to improve control of crop diseases (Pinter et al., 2003; Oerke et al., 2014). This idea is based on approaches of precision farming, where site-specific management of fertilizer and fungicide applications are performed based on input from remote sensing and geographic information systems (Pinter et al., 2003; Steddom et al., 2005; Mattupalli et al., 2015).

Advanced remote sensing techniques enhance the possibility for detecting crop diseases in a rapid and non-invasive way (Mahlein, 2016; Wakie et al., 2016). Yet, there remains a lack of investigations and/or applications of these technologies to advance breeding by improving the efficiency of selection in large, genetically diverse plant populations under field conditions (Deery et al., 2014; Walter et al., 2015). Advances in breeding for disease resistance are often limited by a lack of available resistant varieties and can also be constrained by deficiencies associated with conventional phenotyping methods—for instance, the precision, reproducibility, and throughput of conventional visual assessments (Steddom et al., 2005; O'Driscoll et al., 2014; Mahlein, 2016). To better meet the specific needs of crop breeders, remote, and proximal sensing techniques will need to generate accurate plant phenotypes in genetically diverse populations under field conditions (Furbank and Tester, 2011; Kirchgessner et al., 2017; Yang et al., 2017).

Hyperspectral remote sensing has been shown to be a promising tool for monitoring foliar diseases over scales ranging from individual leaves to fields to entire regions (Mahlein et al., 2013; Ashourloo et al., 2014b; Wakie et al., 2016; Yuan et al., 2016). For instance, hyperspectral reflectance measurements of wheat leaves were successfully used to detect leaf rust and estimate disease severity (Ashourloo et al., 2014a,b). Hyperspectral imaging was successfully used for the detection of *Fusarium* head blight in wheat grains (Bauriegel and Herppich, 2014). For field crops, foliar diseases often occur simultaneously at different leaf layers and sometimes infections begin in the lower canopy. Canopy-level hyperspectral signatures responding to certain disease symptoms can be complicated by crop morphology and canopy structure (Zarco-Tejada et al., 2001; Yu et al., 2014; Mahlein, 2016). Therefore, selection of spectral features that can qualitatively and quantitatively measure a disease irrespective of the different canopy structures likely to exist across a large and diverse population is critical in order for hyperspectral remote sensing to be useful for a plant breeding program.

Some leaf diseases can cause losses of chlorophyll and other diseases may trigger leaf water deficits (Delalieux et al., 2009; Yu et al., 2014; Oerke et al., 2016). As a result, infected plants may present different characteristics at the chlorophyll and/or water absorption bands of their spectral signatures compared to healthy plants. Canopy-level sensing methods and spectral features should be compared for their ability to detect these differing signatures of infection under field conditions. It will be particularly useful to better understand the capabilities of different spectral features to detect and quantify common crop diseases in large and genetically diverse breeding populations that possess diverse canopy structures.

Disease detection can be based on vegetation indices that use approximation methods to quantify leaf biochemical changes, e.g., changes in leaf pigments and water content (Bürling et al., 2011; Ashourloo et al., 2014a; Yu et al., 2015). Hyperspectral narrow vegetation indices enhance the power to detect plant diseases by using high resolution narrow bands that enable characterization of subtle differences in plants and canopies (Delalieux et al., 2009; Mahlein et al., 2013). For instance, hyperspectral disease severity indices have been developed specifically for the detection of wheat leaf rust (Ashourloo et al., 2014a). Different plant diseases can also produce disease-specific spectral signatures that allow for the discrimination of different diseases (Mahlein et al., 2013). For disease resistance breeding, an accurate measure of disease infection across a large and genetically diverse population is critical. Therefore, it is important to know which spectral indices have the best capability to provide an accurate measure of a crop disease. Comparisons of spectral features and spectral indices for crop diseases will also advance sensor development for field-based plant phenotyping and precision disease management.

The objective of this study was to investigate the potential of canopy-level hyperspectral measurements to detect and accurately measure STB infection in a genetically diverse population composed of 335 elite winter wheat varieties, and to determine suitable hyperspectral vegetation indices and the most sensitive spectral features for STB detection and quantification under field conditions.

## Materials and methods

### Plant materials and experimental design

#### Main experiment

A field experiment was conducted during the 2015/2016 winter wheat growing season. The experiment was conducted on a 1 ha field site of the ETH Field Experiment Station Lindau-Eschikon, Switzerland (Kirchgessner et al., 2017). A total of 335 European wheat varieties selected from the GABI-wheat panel (Kollers et al., 2013) were grown in two blocks (replications). Within each block, each variety was grown in a randomly located plot (1.2 × 1.7 m), except for the check plots (grown as controls for field variability) that were planted in 21 evenly distributed locations at which the wheat variety CH CLARO was planted. The sowing and harvest dates were 13 October 2015 and 28 July 2016, respectively.

Fertilization and fungicide applications in the main experiment followed the local recommendations. Fungicide applications were made three times during the growing season with applications on 6 April (BBCH 31; Input® containing prothioconazol and spiroxamine, Bayer CropScience Ltd, Germany), 25 May (BBCH 51; Aviator Xpro containing bixafen and prothioconazole, Bayer CropScience Ltd, Germany) and 6 June (BBCH 65, Osiris® containing epoxiconazol and metconazol, BASF, Germany). Details of fertilizer and fungicide applications were reported in Karisto et al. (2018). In this study, for the purpose of binary classification, the main experimental plots treated with fungicides were considered *healthy* (*Z. tritici un-treated* canopies and referred to as the treatment T0, see next section for T1), though some of these plots were later shown to develop significant levels of STB (Karisto et al., 2018). All STB infections in the T0 plots were natural, a result of the ascospores that immigrated into the experimental plots from wheat fields in the same region. The precipitation between April and end of July was 573 mm, 36% above the reference period 1981–2010 (MeteoSchweiz, 2016). This high precipitation was conducive to development of a strong STB epidemic.

#### Septoria tritici blotch (STB) reference experiment

The STB reference experiment was located next to the blocks of the main experiment. Four varieties (CH CLARO, DRIFTER, RUNAL, and TITLIS), included also in the set of 335 varieties of T0, were used for the *disease* treatment (*Z. tritici* treated, hereafter referred to as T1). As shown in Karisto et al. (2018), DRIFTER is very susceptible, CH CLARO and RUNAL have intermediate resistance, and TITLIS is resistant to STB. Specifically, DRIFTER ranked 2/335 (the 2nd most susceptible variety), CH CLARO ranked 108/335 (moderately susceptible/resistant), RUNAL ranked 151/335 (moderately susceptible/resistant), and TITLIS ranked 305/335 (resistant). The four STB reference varieties were grown in four 2.45 × 3 m plots with two replications, in the absence of fungicide protection. Four Swiss strains of *Z. tritici* were inoculated into these plots on 10 May, 2016 in central areas (0.5 × 0.5 m) of each plot by spraying the whole canopy until runoff using 60 ml of a spore suspension containing 10^6^ spores/ml of each strain on each plot. In addition to the STB infection caused by the four inoculated strains, we observed significant STB infection that developed by natural infection from neighboring wheat fields, but we found practically no other diseases (Karisto et al., 2018).

### Canopy spectral measurements

Hyperspectral reflectance measurement was performed across different growth stages (Table 1) over wheat plots using an ASD FieldSpec® 4 spectroradiometor (ASD Inc.) that is equipped with an optical fiber that uses a field of view of 25°. The fiber was mounted on a tripod arm that allows for measuring from a nadir view at a height of 1.5 m above the ground. Canopy reflectance was collected at 1–2 locations for the main experiment plots (T0) and at 3–5 locations for the larger STB disease reference plots (T1), and for each location the spectrum was determined based on the average of 15–25 spectral records. A Spectralon® white reference panel was used for calibration before measuring canopy reflectance, and the calibration was repeated every 10–15 min or immediately following significant changes in light conditions.

**Table 1:** Measurements for canopy spectra, visual assessment (VA), and leaf sample collections (C) for STB metrics.

**Period**	**Healthy (T0) canopy spectra**	**Diseased (T1) canopy spectra**	**Visual assessment (VA)**	**Leaf collection (C) and imagery assessment**	**Growth stage^†^**
Week 0^*^	26-May-16	05-May-16	20-May-16 (VA1)	20-May-16 (C1)	Booting to heading
Week 1	**22-Jun-16**	**22-Jun-16**	21-Jun-16 (VA2)		End of flowering
Week 2	27-Jun-16		29-Jun-16 (VA3)		Early fruiting
	**01-Jul-16**	**01-Jul-16**		04-Jul-16 (C2)	
Week 3	**07-Jul-16**	**07-Jul-16**			End of fruiting
Week 4	**11-Jul-16**	**11-Jul-16**			Early ripening
Week 5	**18-Jul-16**	**18-Jul-16**			Ripening
Week 6	**20-Jul-16**	**20-Jul-16**			

* Week 0 denotes the period at which the second (25 May) and third (6 Jun) fungicide applications were performed, and therefore it was considered as the beginning period of STB infections. Bold font indicates the paired spectral measurements for both healthy and diseased plots.

† *Growth stage was estimated based on the T0 plots in the main experiment*.

### STB disease assessment

#### Leaf level imagery assessment

Leaves exhibiting obvious STB lesions were sampled from each cultivar on 20 May 2016 (Collection 1, see Table 1) and 4 July 2016 (Collection 2) (Karisto et al., 2018). In each plot of the main experiment, 16 infected leaves were collected, transported to the laboratory in cooled boxes, imaged with flatbed scanners, and analyzed using the method described in Stewart et al. (2016) and Karisto et al. (2018). In this study, the percentage of leaf area covered by lesions (PLACL), the density of pycnidia per unit leaf area (ρ-leaf) and the density of pycnidia per unit lesion area (ρ-lesion) were used as quantitative measures of STB leaf infections.

#### Canopy level visual assessment

Visual assessment of STB symptoms was performed on 20 May, 21 June and 29 June 2016 (Table 1). STB severity in each plot was scored from 1 to 9 (1 and 9 denote no disease and complete infection, respectively), following a logistic (non-linear) progression based on the symptoms of the top three leaf layers (Michel, 2001). This assessment is the standard procedure for identifying STB-resistant germplasm to use for resistance breeding. In our study, the scores ranged only between 1 and 5 in the main experiment as a result of the fungicide treatments; meaning that only mild to moderate STB infections were observed. Based on visual assessments (VA) of STB at three time points (Table 1), the area under disease progress curve (AUDPC) was calculated for each variety (Karisto et al., 2018).

### Data analysis

Canopy reflectance values for individual plots were averaged and served as input data for the calculation of hyperspectral narrow-band indices based on the formulae listed in Table 2. Binary logistic regression (BLR) was employed for the evaluation of each spectral index for the detection of plant diseases at different time points of the STB epidemic. The c-statistic of spectral indices based BLR models can be used as a measure to assess the discriminatory performance of spectral indices (Delalieux et al., 2009; Yu et al., 2014). We calculated the c-statistic for each spectral index in detecting the event of interest (disease) based on the four reference varieties. The c-statistic value is equivalent to the area under the receiver-operating-characteristic (ROC) curve. We used a general rule that considers: 0.7 ≤ c < 0.8 as acceptable discrimination; 0.8 ≤ c < 0.9 as excellent discrimination; and c ≥ 0.9 as outstanding discrimination (Hosmer and Lemeshow, 2005).

**Table 2:** Hyperspectral vegetation indices (dimensionless) used in this study for wheat STB disease detection.

**Index**	**Description**	**Application**	Formula^*^	**References**
SR_Red−edge_	Simple ratio at red edge	Chlorophyll	R750/R710	Zarco-Tejada et al., 2001
CI_Red−edge_	Chlorophyll index at red edge	Chlorophyll	(R750 – R700)/R700	Gitelson et al., 2003
NDVI	Normalized difference vegetation index	Green biomass, LAI	(R800 – R680)/(R800 + R680)	Sims and Gamon, 2002
ND705	Normalized difference at 705 nm	Chlorophyll	(R750 – R705)/(R750 + R705)	Sims and Gamon, 2002
mSR705	Modified simple ratio at 705 nm	Chlorophyll	(R750 – R445)/(R705 – R445)	Sims and Gamon, 2002
mND705	Modified ND705	Chlorophyll	(R750 – R705)/(R750 + R705 – 2^*^R445)	Sims and Gamon, 2002
SIPI	Structure insensitive pigment index	Pigment ratio between carotenoid and chlorophyll *a*	(R800 – R445)/(R800 – R680)	Peñuelas et al., 1995b
PRI	Photochemical reflectance index	Physiology, photosynthesis	(R531 – R570)/(R531 + R570)	Gamon et al., 1997
WI	Water index	Water content	R900/R970	Peñuelas and Inoue, 1999
NDWI	Normalized difference water index	Water content	(R857 – R1241)/(R857 + R1241)	Gao, 1996
PSRI	Plant senescence reflectance index	Senescence	(R678 – R500)/R750	Merzlyak et al., 1999
RRDI_Red−edge_	Ratio of reflectance difference index at red edge	Chlorophyll	(R745 – R740)/(R740 – R700)	Yu et al., 2015
NPQI	Normalized phaeophytinization index	Disease, chlorophyll degradation	(R415 – R435)/(R415 + R435)	Peñuelas et al., 1995a
CAI	Chlorophyll absorption integral	Disease, chlorophyll	$\int_{600}^{740}R\left(EQ\right)$	Oppelt, 2002; Laudien et al., 2003
HI	Healthy index	Disease	(R534 – R698)/(R534 + R698) – $\frac{1}{2}$^*^R704	Mahlein et al., 2013
DSI	Leaf rust disease severity index	Disease	6.9^*^(R605 – R455) – 1.2	Ashourloo et al., 2014a

* *Ri denotes the reflectance at wavelength i nm; EQ = Ri/Ei, where Ei denotes the envelope (straight line connecting 600 and 740 nm) reflectance at wavelength i nm*.

Spearman's correlation analysis was applied to study the relationships between the spectral indices and STB measurements to assess their monotonic relationships (whether linear or not). We used partial least squares (PLS) regression (PLSR) to characterize the STB infection severity in the full spectrum of 350–2,500 nm, as well as partial least squares discriminant analysis (PLSDA) for the differentiation between diseased and healthy plots. PLS is a multivariate method which relates two matrices, X and Y, i.e., explanatory and response matrices, by extracting a number of components (also known as latent variables) to model the variations of both matrices (Wold et al., 2001). The variable importance in projections (VIP) of PLS models were computed to evaluate the importance of individual bands in modeling STB metrics, and VIP ≥ 0.8 was considered as the threshold for important contributions (Wold et al., 2001; Eriksson et al., 2013). The optimal number of components was determined by finding the lowest root mean squared error of prediction (RMSEP) and predicted residual sum of squares PRESS (van der Voet, 1994).

Spectral data for the four STB disease reference varieties (CH CLARO, DRIFTER, RUNAL, and TITLIS) were used to calibrate the BLR and PLSDA models. The remaining 331 varieties were used as independent validation data to test the classification performance. The sampling periods in which STB metrics were best correlated (Week 3, Table 1) with canopy spectra were used for the calibration and comparison of classification models. The validation dataset also includes data of the four reference varieties in Week 2, which is used to test the capability of early detection of STB infections.

Classification performance is illustrated in a confusion matrix with the following measures: $Sensitivity=\frac{TP}{\left(TP+FN\right)}$ (true positive rate, TPR), $Specificity=\frac{TN}{\left(TN+FP\right)}$ (true negative rate, TNR), $Accuracy=\frac{\left(TP+TN\right)}{\left(TP+TN+FP+FN\right)}$, where *T, F, P*, and *N* denote *true, false, positive* and *negative*, respectively, and the Kappa statistic. The Kappa statistic measures the agreement (1 represents perfect agreement) between the actual (the assigned treatments) and model predicted classes, and it accounts for chance agreement (McHugh, 2012). Hence, it is a balanced measure for unbalanced classification problems as in this study where we have a much smaller sample size for the diseased treatment.

(1) $$\begin{array}{l} Kappa=\frac{p_{a}-p_{e}}{1-p_{e}} \end{array}$$

(2) $$\begin{array}{l} p_{e}=\frac{\left(TN+FP\right)\times \left(TN+FN\right)+\left(FN+TP\right)\times \left(FP+TP\right)}{{\left(TP+TN+FP+FN\right)}^{2}} \end{array}$$

where *p*_*a*_ is the actual agreement (identical to accuracy), whereas *p*_*e*_ is the expected agreement by chance (random accuracy) that can be calculated as Equation (2). Letters *T* and *F* used in the equation terms denote *true* and *false*, respectively, and *P* and *N* denote *positive* and *negative*, respectively.

All analyses were performed in the R environment (R Core Team, 2016). PLSR was implemented using the r package “pls” (Mevik and Wehrens, 2007). PLSDA was implemented using the package “mixOmics” (Rohart et al., 2017).

## Results and discussion

### Canopy spectral characteristics of STB infection

**Figure 1:**
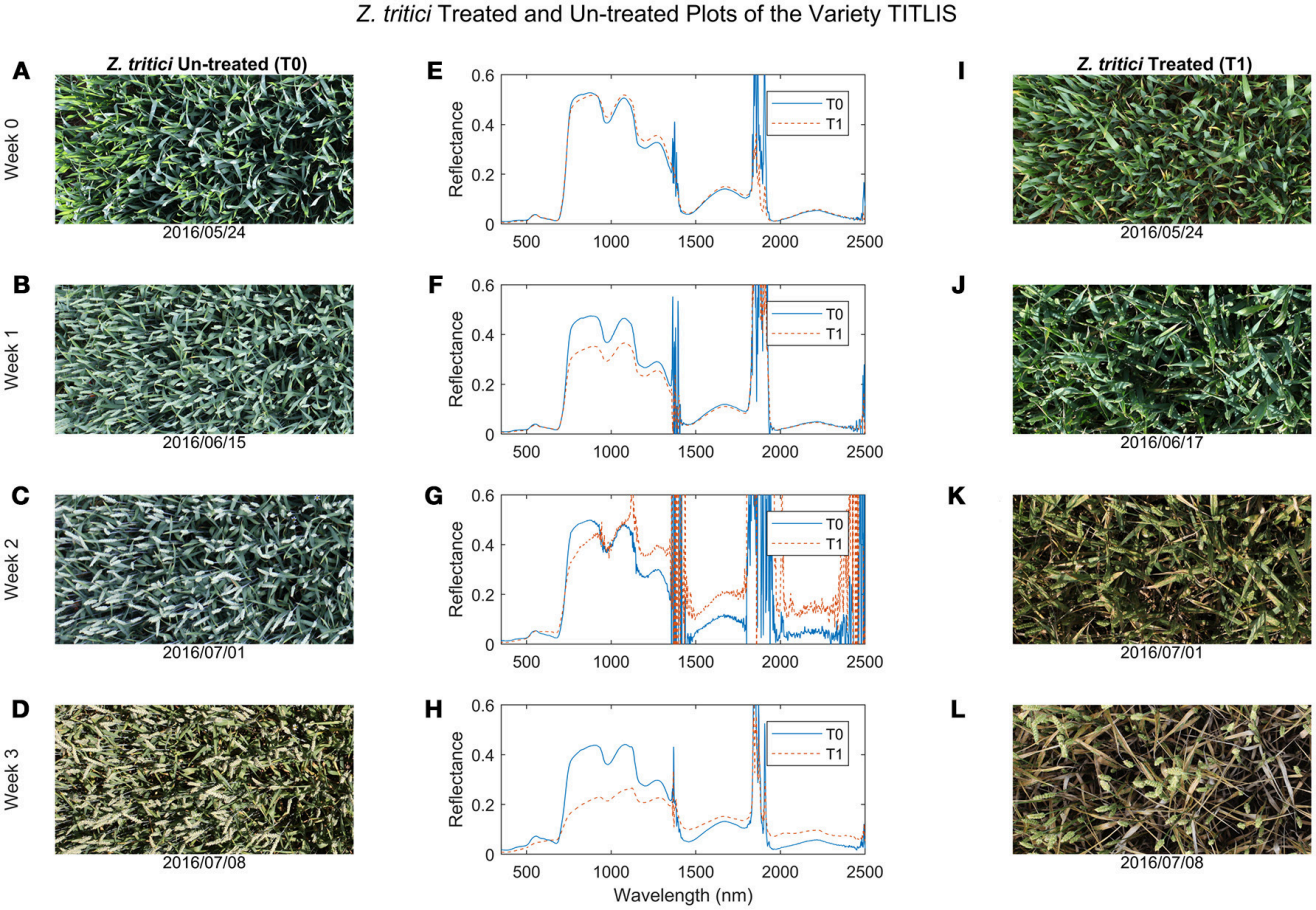
Comparisons of wheat canopy RGB images and hyperspectral reflectance curves between the *Zymoseptoria tritici* un-treated (T0, images **I–L**) and treated (T1, images **A–D**) plots at four periods. **(A,E,I**) Week 0 was at the end of May 2016; **(B,F,J**) Week 1 was at mid-June 2016; **(C,G,K**) Week 2 was at the beginning of July; and **(D,H,L**) Week 3 was at mid-July 2016 (see also Table 1 for a full list of sampling periods and corresponding growth stages).

At the early stage of the STB infection, only marginal differences were visible between the diseased and healthy canopies (Figures 1A,I) and there were no distinct differences in the canopy reflectance (Figure 1E). As the epidemic progressed, STB symptoms became obvious in the diseased canopies (Figures 1B–D,J–L) and differences in canopy reflectance between diseased and healthy plots of the same variety became more pronounced (Figures 1F–H).

**Figure 2:**
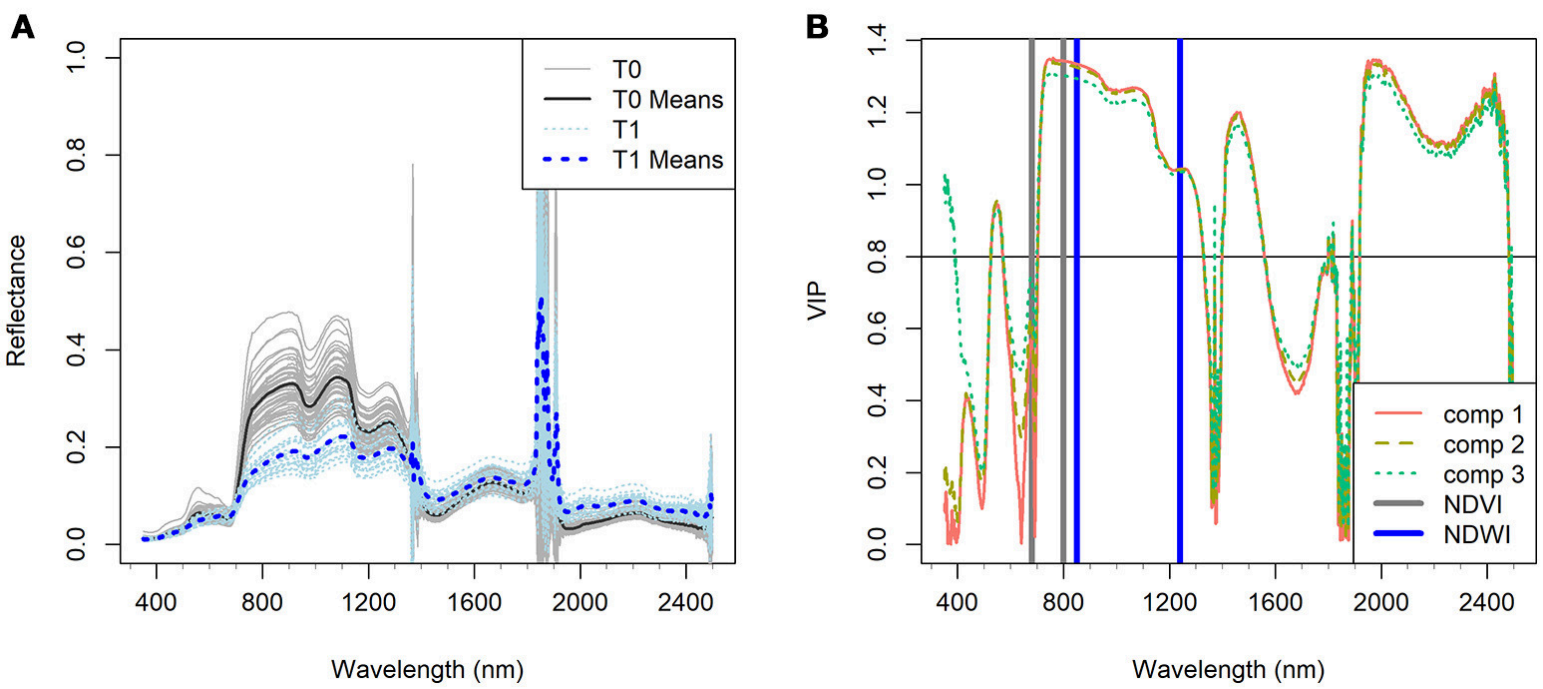
**(A)** Canopy hyperspectral reflectance and reflectance means of the *Zymoseptoria tritici* treated wheat plots (T1) in comparison with the Z. *tritici* untreated plots (T0) of the four reference varieties (CH CLARO, DRIFTER, RUNAL and TITLIS) at the stage of significant infection (Week 3). **(B)** Variable importance in projections (VIP, dimensionless) of the first three components (comp 1, 2, and 3) of PLSDA model in characterizing the difference of the two treatments (VIP ≥ 0.8 is considered as the threshold of influential contribution). Gray and blue bars indicate the spectral bands of NDVI and NDWI respectively (see formulae in Table 2).

By the late stage of the STB epidemic, canopy reflectance in the green region was much lower for the diseased plots than for the healthy plots (Figure 1H), whereas reflectance in the red region was higher in diseased compared to healthy plots (Figures 1G,H, 2A). Reflectance in the green and red regions is associated mainly with green canopy fraction and leaf pigments (Ustin et al., 2009). Plant diseases often cause chlorotic and necrotic leaf surfaces that lack chlorophyll and thus affect leaf and canopy reflectance and transmittance in the red and green regions (Oerke et al., 2016). Therefore, the reflectance changes in the visible region observed in the diseased canopies suggest that STB infections cause reductions in leaf chlorophyll and corresponding reductions in photosynthetic activity (Mahlein, 2016; Oerke et al., 2016).

Reflectance in the NIR (750–1,300 nm) region was distinctly lower in the diseased canopies than in the healthy canopies at week 3 (Figure 2A). Reflectance in the NIR is governed mainly by the internal leaf structure as well as by the structure of the whole canopy. It appears that STB infection caused damage to the internal and surface structures of leaves that increased water loss, resulting in a significant decrease in NIR reflectance in the diseased canopies (Figure 2A). The STB-induced canopy spectral signature also showed typical vegetation stress characteristics in the shortwave infrared (SWIR, 1,300–2,500 nm) regions, where reflectance in the diseased plots was higher than in the healthy plots (Figures 1G,H, 2A). Leaf reflectance in the SWIR region is associated with water absorption as well as proteins and carbohydrates (Kumar et al., 2001; Thenkabail et al., 2011), suggesting that STB infections decreased the water status of the diseased canopy leaves leading to low absorption and high SWIR reflectance. However, the observed canopy spectral responses in the chlorophyll and water absorption regions might be similar to those caused by other diseases or stresses. The changing spectral characteristics over time associated with the development of particular diseases or stresses may make it possible to identify the particular causes of stress by measuring canopy spectra across several time points.

VIP scores provide a measure of the importance of variables used in PLS modeling, and therefore the VIP scores over the full spectrum indicate the contribution of spectral bands in characterizing the response variables, i.e., the STB infection metrics used in this study. Figure 2B highlights the predominant contribution (VIP ≥ 0.8 is considered as influential contribution) of the NIR region and water absorption bands in differentiating between the healthy and diseased canopies. Healthy canopies yielded low reflectance and high absorption in the water absorption bands in the NIR and SWIR regions. This is attributed to the fact that healthy leaves have more compact leaf mesophyll layers and fewer air-water interfaces than diseased leaves, resulting in decreased reflectance in the NIR region (Kumar et al., 2001).

### Spectral indices for STB detection

#### Correlation between canopy STB metrics and spectral indices

**Figure 3:**
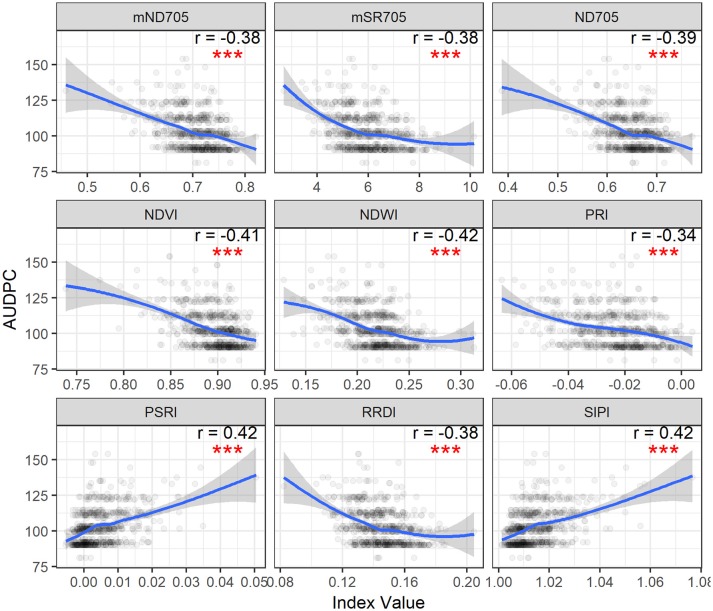
Area under disease progress curve (AUDPC) of visual scores plotted as a function of spectral indices. Correlation coefficients (*r*) are for the Spearman's correlations between the spectral indices and AUDPC of STB scores (^***^p < 0.001). Loess fit lines are added to the scatter plot to show the trend of the relationship. Results are based on the data of the whole population (335 varieties) used in the main experiment.

Spearman's correlation was measured between canopy STB metrics (STB scores and AUDPC) and spectral indices (Table 3, Figure S1 and Figure 3). STB scores correlated positively with PSRI and SIPI, whereas they were negatively related to other spectral indices. The correlation coefficients for the third assessment (VA3) were larger than for the two earlier assessments, consistent with the continuous increase in STB infections. As expected, AUDPC yielded higher correlation with the spectral indices than the STB scores for individual assessments. STB scores and AUDPC both resulted in positive correlations with PSRI and negative correlations with NDWI (Figure S1 and Figure 3), suggesting associations between the amount of STB infection and the degree of leaf senescence and water status. Other studies reported that diseased leaves are prone to suffer severe water deficits as a result of stomatal opening disorders (Oerke et al., 2016), and water deficit has been shown to play a key role in accelerating leaf senescence (Rivero et al., 2007). We hypothesize that this explains the observed positive correlations between PSRI and the STB visual assessments.

**Table 3:** Correlation coefficients of the Spearman's correlations between the spectral indices and the two canopy measures (visually assessed STB scores and corresponding AUDPC values) at three visual assessments (VA1, VA2, and VA3) and the three leaf STB metrics (PLACL, ρ-leaf, and ρ-lesion) at two collections (C1 and C2) of leaf samples.

**Index**	**STB score**			**AUDPC**	**PLACL**		ρ**-lesion**		ρ**-leaf**	
	**VA1**	**VA2**	**VA3**	**VA1-3**	**C1**	**C2**	**C1**	**C2**	**C1**	**C2**
SR_Red−edge_	−0.03	−0.2^***^	−0.23^***^	−0.39^***^	−0.35^***^	−0.54^***^	0.17^***^	−0.19^***^	−0.25^***^	−0.49^***^
CI_Red−edge_	−0.03	−0.21^***^	−0.24^***^	−0.39^***^	−0.37^***^	−0.54^***^	0.17^***^	−0.20^***^	−0.27^***^	−0.50^***^
NDVI	−0.03	−0.19^***^	−0.24^***^	−0.41^***^	−0.38^***^	−0.55^***^	0.16^***^	−0.22^***^	−0.28^***^	−0.52^***^
ND705	−0.03	−0.2^***^	−0.23^***^	−0.39^***^	−0.36^***^	−0.54^***^	0.17^***^	−0.20^***^	−0.26^***^	−0.50^***^
mSR705	−0.04	−0.21^***^	−0.23^***^	−0.38^***^	−0.35^***^	−0.54^***^	0.15^***^	−0.19^***^	−0.26^***^	−0.50^***^
mND705	−0.04	−0.21^***^	−0.23^***^	−0.38^***^	−0.35^***^	−0.54^***^	0.15^***^	−0.19^***^	−0.26^***^	−0.50^***^
SIPI	0.10^***^	0.27^***^	0.27^***^	0.42^***^	0.22^***^	0.56^***^	0.04	0.21^***^	0.23^***^	0.53^***^
PRI	−0.01	−0.28^***^	−0.23^***^	−0.34^***^	−0.14^***^	−0.53^***^	0.03	−0.14^***^	−0.11^***^	−0.46^***^
WI	0.02	0.15^***^	0.16^***^	0.29^***^	0.26^***^	0.54^***^	−0.09^**^	0.22^***^	0.24^***^	0.51^***^
NDWI	−0.05	−0.22^***^	−0.24^***^	−0.42^***^	−0.28^***^	−0.56^***^	0.06^*^	−0.24^***^	−0.27^***^	−0.55^***^
PSRI	0.08^**^	0.27^***^	0.27^***^	0.42^***^	0.10^***^	0.57^***^	0.11^***^	0.20^***^	0.17^***^	0.53^***^
RRDI_Red−edge_	−0.06^*^	−0.21^***^	−0.21^***^	−0.38^***^	−0.36^***^	−0.47^***^	0.13^***^	−0.15^***^	−0.31^***^	−0.42^***^
NPQI	0.02	−0.02	−0.04	−0.04	0.05	−0.11^**^	−0.09^**^	−0.05	−0.03	−0.11^**^
CAI	−0.02	−0.12^**^	−0.22^***^	−0.31^***^	0.09^**^	−0.55^***^	−0.13^***^	−0.24^***^	0	−0.55^***^
HI	−0.05	−0.28^***^	−0.25^***^	−0.38^***^	−0.24^***^	−0.54^***^	−0.01	−0.15^***^	−0.24^***^	−0.47^***^
DSI	0.02	0.16^***^	0.12^**^	0.24^***^	0.28^***^	0.30^***^	−0.16^***^	0.09^*^	0.18^***^	0.25^***^

* p < 0.05;

** p < 0.01;

*** *p < 0.001; otherwise, not significant*.

STB scores and AUDPC were negatively correlated to the greenness- or chlorophyll-related spectral indices such as NDVI, ND705, mSR705, and CAI, in agreement with studies that reported negative correlations between plant disease severity and NDVI and visible band spectral indices (Steddom et al., 2005; Ashourloo et al., 2014a; Jansen et al., 2014). In contrast, SIPI was positively related to the STB scores and AUDPC despite using the visible bands related to plant pigments. SIPI estimates the pigment ratio between carotenoid and chlorophyll *a* (Car/Chl*a*) and its increase is often related to stresses and subsequent changes in physiological and phenological status (Peñuelas et al., 1995b). Similarly, positive correlations have also been reported between the SIPI and visual rating scores of soybean diseases (Bajwa et al., 2017). Therefore, SIPI shows the potential of being a surrogate of plant disease severity under field conditions.

#### Correlation between leaf STB metrics and spectral indices

Table 3 shows the correlation coefficients for the relationships between the three leaf-based STB metrics (PLACL, ρ-leaf, and ρ-lesion) and the spectral indices for the two collections. The correlation coefficients for the second collection were higher than for the first collection, which is in agreement with the results of the visual assessments and confirms the continuous increase in STB infections during the epidemic. Compared to the first collection, PLACL and ρ-leaf doubled their correlation coefficients in the second collection. In contrast, the correlation for ρ-lesion increased only marginally compared to the first collection. These results indicate that the canopy level spectra might be of limited value for detecting subtle differences associated with the number of pycnidia occurring within lesions.

**Figure 4:**
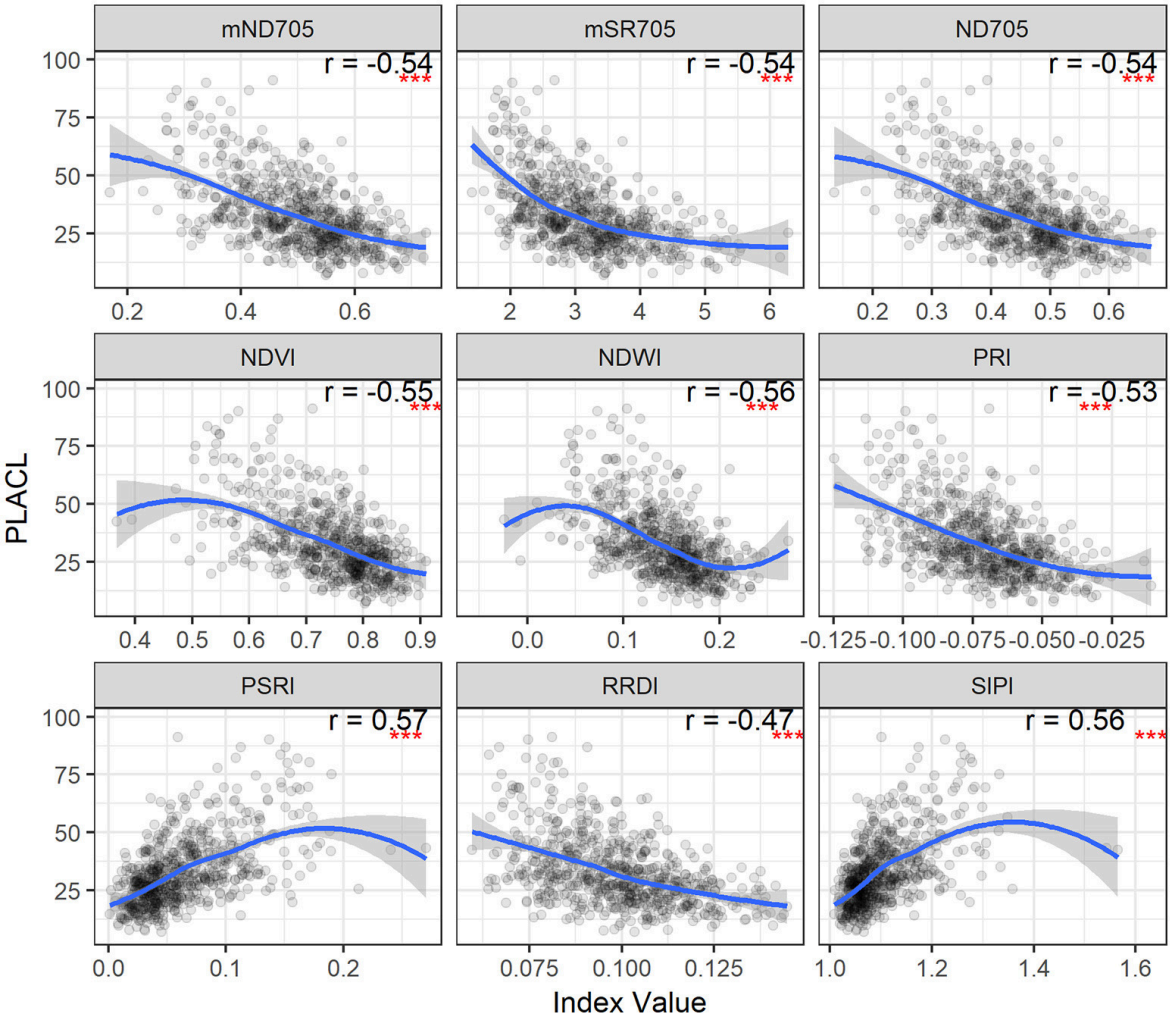
STB percentage of leaf area covered by lesions (PLACL) plotted as a function of spectral indices for the second collection. Correlation coefficients (*r*) are for the Spearman's correlations between the spectral indices and PLACL (^***^p < 0.001). Loess fit lines are added to the scatter plot to show the trend of the relationship. Results are based on the data of the whole population (335 varieties) used in the main experiment.

**Figure 5:**
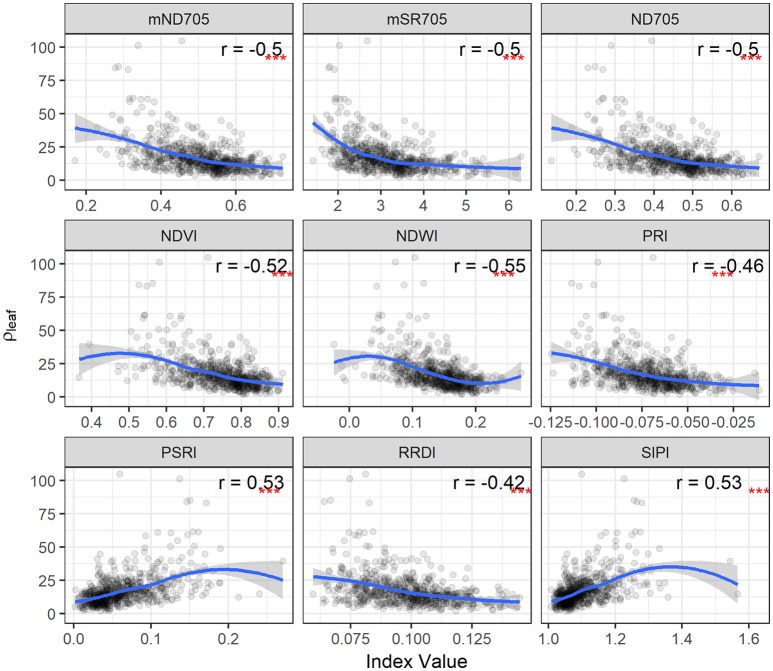
STB density of pycnidia per unit leaf area (ρ_leaf_) plotted as a function of spectral indices for the second collection. Correlation coefficients (*r*) are for the Spearman's correlations between the spectral indices and ρ_leaf_ (^***^p < 0.001). Loess fit lines are added to the scatter plot to show the trend of the relationship. Results are based on the data of the whole population (335 varieties) used in the main experiment.

The relationships between the spectral indices and leaf STB metrics for the second collection are illustrated in Figures 4, 5 and Figure S2. The spectral indices correlating negatively with PLACL are mainly related to vegetation fraction (e.g., NDVI), water content (NDWI), and chlorophyll or photosynthetic capacity (e.g., ND705, PRI). In contrast, PSRI and SIPI were positively related to the PLACL (Figure 4). Similar to the canopy STB metrics, SIPI was positively related to PLACL, ρ-leaf, and ρ-lesion, suggesting that the physiological and/or phenological changes in diseased leaves can be captured in hyperspectral signatures at both leaf and canopy levels (Peñuelas et al., 1995b; Yang et al., 2007; Bajwa et al., 2017). Here, increases in SIPI and PSRI were associated with increased degrees of lesion development on leaves, which resulted in decreased photosynthetic activities and also enhanced the senescence-related signatures that were observed from the canopy.

For PLACL and ρ-leaf, PSRI and SIPI yielded the same magnitude of correlations (Figures 4, **5**). However, the correlations were significantly weaker for the relationships with the ρ-lesion (Figure S2). PLACL is the best indicator of STB damage to the host plant because it directly measures the loss in photosynthetic area due to infection, whereas ρ-lesion is the best indicator of pathogen reproduction on the host plant because it is a direct measure of the ability of the pathogen to convert infected plant tissue into pathogen reproductive structures. The combined measure ρ-leaf provides the best overall measurement of relative resistance to STB (Karisto et al., 2018). Our results suggest that canopy-level hyperspectral indices may be less useful for identifying resistance that affects pathogen reproduction (i.e., ρ-lesion), but could be very useful for identifying resistance that affects host damage (i.e., PLACL). Further improvements may be possible by applying high spatial resolution hyperspectral imaging sensors (Mahlein, 2016; Oerke et al., 2016).

Overall, consistent trends (either positive or negative relationships) were observed in the correlations between canopy spectral indices and both canopy- and leaf-level STB metrics. Spectral indices related to chlorophyll and water status were always negatively related to STB metrics, whereas the senescence-related indices were always positively related. Our results suggest that canopy hyperspectral measurements can provide a quantitative measure of STB damage on wheat and provide a useful complement to traditional visual disease ratings.

#### Discriminant analysis using spectral indices

**Figure 6:**
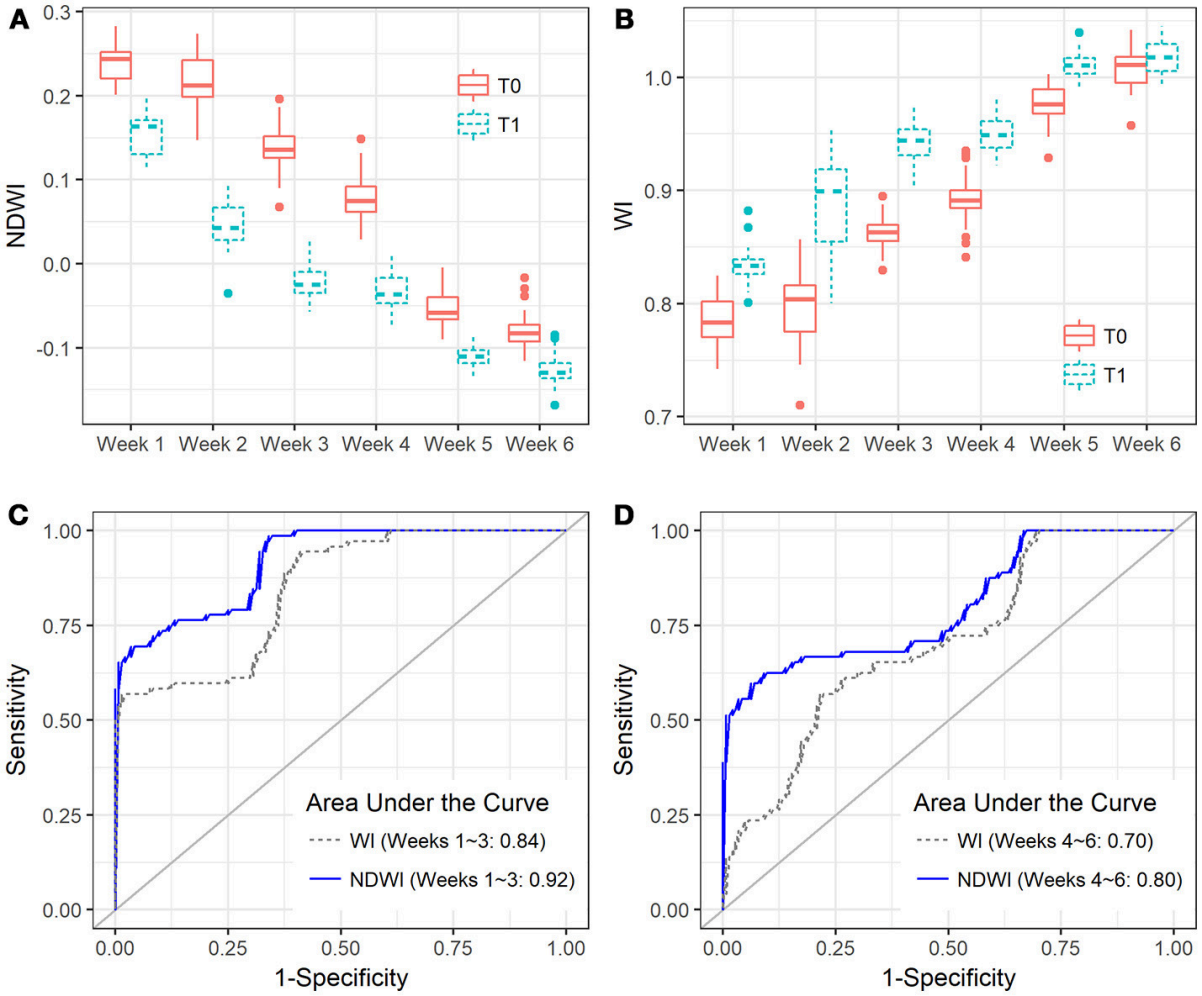
Boxplots show the capability of **(A)** NDWI and **(B)** WI in discriminating between the *Zymoseptoria tritici* un-treated (T0) and treated canopies (T1) of the four reference varieties (CH CLARO, DRIFTER, RUNAL, and TITLIS) in different sampling periods, and ROC plots show the comparison of NDWI and WI at the early (**C**, Weeks 1–3) and late (**D**, Weeks 4–6) periods.

Spectral indices are also used to discriminate stressed or diseased plants from healthy ones. Thus, understanding which spectral indices are suitable for identifying STB resistance is critical. BLR discriminant analysis shows the performances of spectral indices in distinguishing the diseased canopies from the healthy canopies. C-statistic values indicate the capability of spectral indices in the early (weeks 1–3, heading to fruiting stages) and late (weeks 4–6, ripening) periods (Table 4). Water band indices NDWI and WI yielded the best performance for discrimination for the early period, with c-statistics of 0.92 and 0.84, respectively, followed by other indices developed for chlorophyll estimation. Compared to healthy canopies, diseased canopies yielded high NDWI and low WI values at each time point throughout the entire observation period (Figures 6A,B). We also found that spectral indices performed differently in early (weeks 1–3, heading to fruiting stages) and late periods (weeks 4–6, ripening) of the STB epidemic (Table 4 and Figures 6C,D). Most of the spectral indices performed better in the early periods than in the late periods, except for the NPQI and DSI that showed better performances in the late periods. The presence of the spikes might contribute to the different performances of spectral indices at different sampling periods, since different indices and use of different spectral bands can have varied sensitivity to canopy structure changes due to spike presence.

**Table 4:** The c-statistic of logistic regression model showing the performance of spectral indices in discriminating between the healthy (T0) and disease (T1) treatments of the four reference varieties [bold font highlights c-values ≥ 0.8 for the early (Weeks 1–3, heading to fruiting stages) and later stages (Weeks 4–6, ripening stage)].

**Index**	**C-statistic**		
	**Weeks 1–3**	**Weeks 4–6**	**All**
SR_Red−edge_	0.77	0.54	0.59
CI_Red−edge_	0.78	0.54	0.59
NDVI	0.79	0.58	0.61
ND705	0.77	0.54	0.59
mSR705	0.77	0.51	0.58
mND705	0.77	0.51	0.58
SIPI	**0.80**	0.55	0.61
PRI	0.77	0.71	0.54
WI	**0.84**	0.70	0.68
NDWI	**0.92**	**0.80**	0.74
PSRI	**0.82**	0.43	0.58
RRDI_Red−edge_	0.72	0.60	0.57
NPQI	0.70	**0.83**	0.70
CAI	**0.83**	0.61	0.64
HI	**0.80**	0.76	0.54
DSI	0.62	0.79	0.56

NDWI was the best index for detecting STB disease (Table 4 and Figures 6C,D), suggesting new insights for developing new crop sensors for early detection of diseases at the field or regional scales. Compared to the VIP scores of NDVI bands, both spectral bands of NDWI yielded significant contributions (VIP >1) in characterizing the differences between healthy and STB diseased canopies (Figure 2B). Until now, most of the hyperspectral cameras carried on near ground and low-altitude platforms (e.g., UAVs) for agricultural applications use only visible and NIR bands (e.g., 400–900 nm) (Garcia-Ruiz et al., 2013; Bareth et al., 2015), which constrains the potential of hyperspectral remote sensing for disease detection and disease mapping due to the absence of water absorption bands.

BLR models based on the selected spectral indices were applied to the independent validation dataset containing 331 varieties in week 3 (fruiting to early-ripening stages) as well as the same varieties in week 2 (early-fruiting stage). Our results indicate that NDWI enabled the best classification when considering sensitivity and specificity together, with an overall accuracy of 95% (kappa = 0.56) (Table 5). Model test sensitivity (i.e., TPR) ranged from 16.7 to 91.7% and test specificity (i.e., TNR) ranged from 37.2 to 97.2% (Table 5). NPQI and HI showed the best ability to correctly identify the diseased canopies, with sensitivities of 91.7 and 70.8%, respectively, whereas NDWI and CAI showed the best ability to correctly identify the healthy canopies, yielding specificities of 97.2 and 95.5%, respectively.

**Table 5:** Confusion matrix of the disease classification using BLR models based on spectral indices and the PLSDA model when applied to the validation dataset (*n* = 758).

**Model**	**Predicted**	**Actual**		**Sensitivity (%)**	**Specificity (%)**	**Overall accuracy (%)**	**Kappa**
		***Healthy* 0**	***Diseased* 1**				
SR_Red−edge_	0	652	40	16.67	91.83	87.07	0.07
	1	58	8				
CI_Red−edge_	0	660	40	16.67	92.96	88.13	0.09
	1	50	8				
NDVI	0	667	40	16.67	93.94	89.05	0.10
	1	43	8				
ND705	0	659	40	16.67	92.82	87.99	0.09
	1	51	8				
mSR705	0	655	40	16.67	92.25	87.47	0.08
	1	55	8				
mND705	0	655	40	16.67	92.25	87.47	0.08
	1	55	8				
SIPI	0	673	40	16.67	94.79	89.84	0.12
	1	37	8				
PRI	0	604	26	45.83	85.07	82.59	0.17
	1	106	22				
WI	0	641	24	50	90.28	87.73	0.28
	1	69	24				
NDWI	0	690	20	58.33	97.18	94.72	0.56
	1	20	28				
PSRI	0	667	34	29.17	93.94	89.84	0.21
	1	43	14				
RRDI_Red−edge_	0	545	30	37.5	76.76	74.27	0.06
	1	165	18				
NPQI	0	415	4	91.67	58.45	60.55	0.13
	1	295	44				
CAI	0	678	34	29.17	95.49	91.29	0.25
	1	32	14				
HI	0	616	14	70.83	86.76	85.75	0.32
	1	94	34				
DSI	0	264	26	45.83	37.18	37.73	−0.03
	1	446	22				
PLSDA	0	658	2	95.83	92.68	92.88	0.60
	1	52	46				

### Full-spectrum analysis for STB detection

#### Estimation of STB metrics using PLSR models

**Figure 7:**
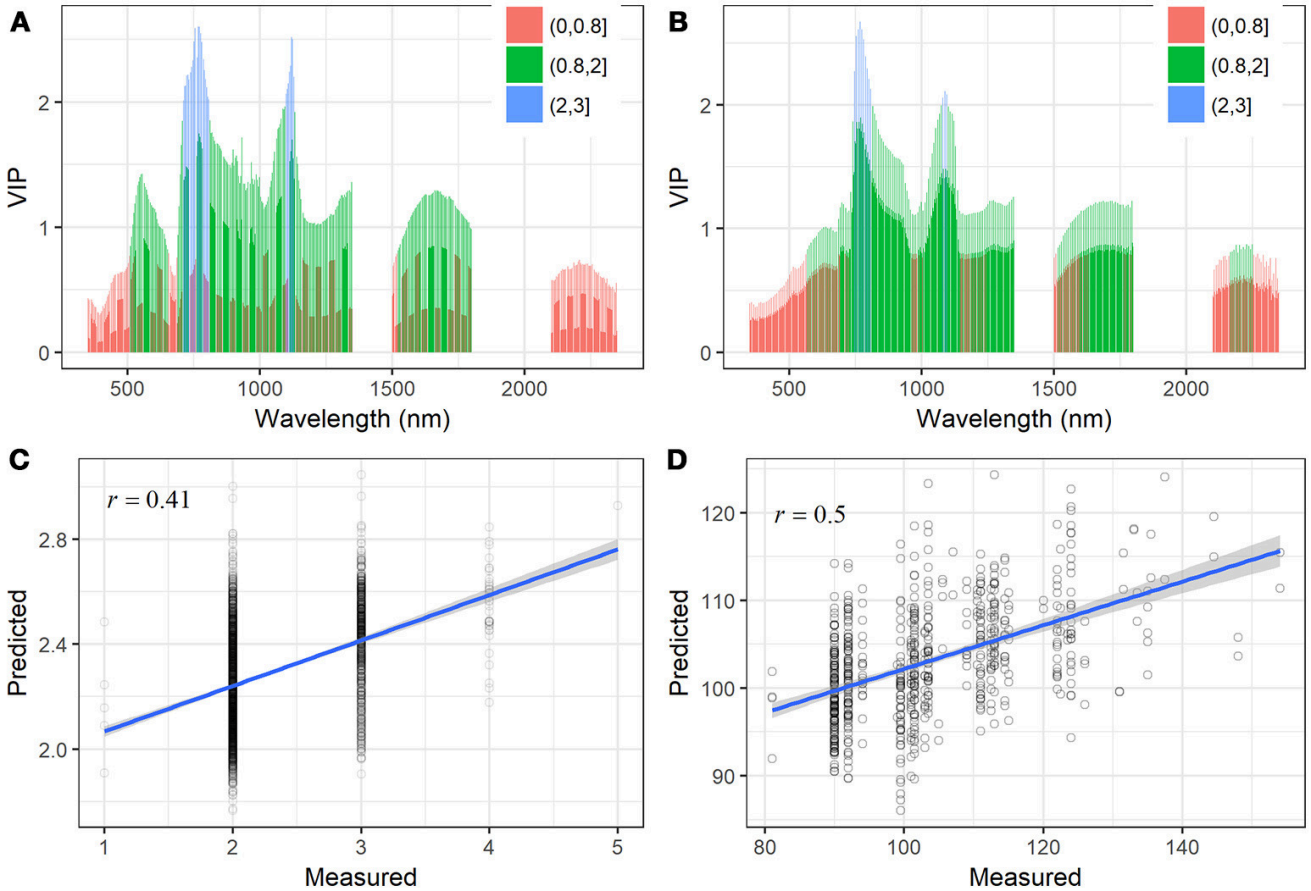
VIP scores of the PLSR models for **(A)** the STB scores and **(B)** AUDPC. The three value breaks show different ranges of VIP values, which indicates the most influential wavelengths. PLSR model predicted values of **(C)** STB scores and **(D)** AUDPC plotted against their measured values with linear fit. Pearson and Spearman correlations produced the same correlation coefficients (*r*).

To further investigate to what extent the full spectrum is able to account for the variations in STB metrics, PLSR models were constructed for the prediction of the STB scores and AUDPC, respectively. The optimal number of components used in the PLSR models for the STB scores and AUDPC was 4 and 3, respectively (Figure S3). The most influential bands (VIP >2) for STB scores and AUDPC were at the red-edge to NIR (700–800 and 1,100–1,150 nm) (Figures 7A,B). Red edge is associated with chlorophyll content and physiological changes due to stresses (Kumar et al., 2001; Sims and Gamon, 2002; Gitelson et al., 2003), which explains the high VIP scores. The VIP method allowed for selection of the spectral bands accounting for the variations in leaf pigment, water status, and internal structural changes. For the sake of comparison with the selected spectral indices, Figure 7 shows the relationships of the predicted STB scores and AUDPC against their measured values, as well as their Spearman's correlations. The PLSR model improved the prediction for STB scores, yielding a correlation coefficient of 0.41 (Figure 7C). Similarly, the PLSR model improved the prediction for AUDPC compared to the best index (SIPI, *r* = 0.42) and yielded an increased correlation coefficient (*r* = 0.50, Figure 7D).

**Figure 8:**
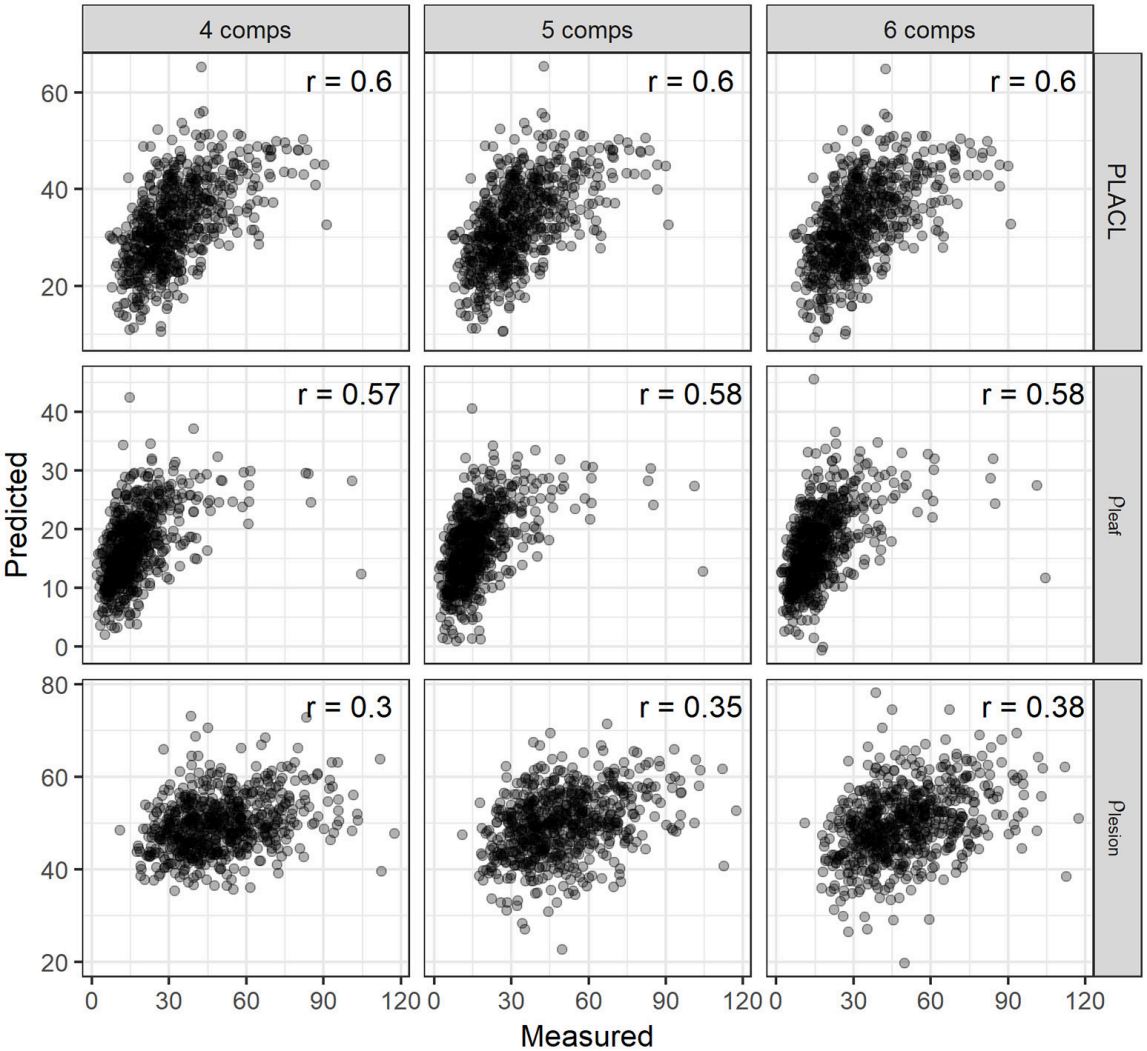
The validation for the correlations between the observed and predicted values of PLACL, ρ-leaf and ρ-lesion using the PLSR model employing the number of components (comps) of 4, 5, and 6. Correlation coefficients (*r*) are for the Spearman's correlations.

For the three leaf-based STB metrics, we constructed a multivariate PLSR model, i.e., with multiple response variables (PLACL, ρ-leaf, and ρ-lesion). To determine the suitable number of components, the PLSR model was first trained with 10 components, and the optimal number of components was determined based on the RMSEP and PRESS statistics (van der Voet, 1994). Results of cross validation showed that the use of four–six components allowed for low error rates and a small number of components (Figure S4). Therefore, the PLSR model for the leaf STB metrics was calibrated by using six components. The PLSR model improved the predictions for leaf STB metrics compared to spectral indices, yielding correlation coefficients of 0.60, 0.58, and 0.38 for PLACL, ρ-leaf, and ρ-lesion respectively (Figure 8).

In contrast to the selected spectral indices that use 2–3 bands, PLSR models make use of the full spectrum and select a subset of important bands that account for the more complete variations in infected canopies. Spectral bands yielding the highest VIP scores (≥ 4) were located in the red edge (730 nm) and NIR (e.g., peaks at 930, 1150 nm) regions (Figure S5), which again suggests the strong association of these spectral bands with STB infections and subsequent physiological changes of plants. Although the PLSR model accounted for smaller variations in the ρ-lesion than in the PLACL and ρ-leaf, the extent of improvement relative to spectral indices was largest for the ρ-lesion (PLSR vs. NDWI).

VI-based modeling would need different indices for individual canopy- or leaf-level STB metrics, suggesting that several individual models may be needed to achieve a certain accuracy (Mahlein et al., 2013; Jansen et al., 2014). In contrast, as observed here, the PLSR model allowed for improvement in linking canopy reflectance signatures to the three leaf STB metrics, PLACL, ρ-leaf, and ρ-lesion, by calibrating one model for multiple response variables (Figure 8). Further investigations of its potential for quantifying multi-infections and diseases are required, especially for the purpose of multiple diseases (Mahlein et al., 2013). Nevertheless, full-spectrum PLSR modeling shows promise for quantifying many traits associated with plant disease infections, e.g., damage to host plants and pathogen reproduction.

#### Classification of healthy and diseased canopies using PLSDA model

Similar to the calibration of BLR models, canopy spectral data of the four reference varieties were used to calibrate the PLSDA model. The PLSDA model was first trained with ten components to determine the optimal number of components for the model, by employing a 5-fold cross-validation to compute the estimation errors. Results showed that the use of four components allowed for low prediction errors whilst maintaining high efficiency of the model (Figure S6), and thus it was decided to use four components for calibrating the model.

**Figure 9:**
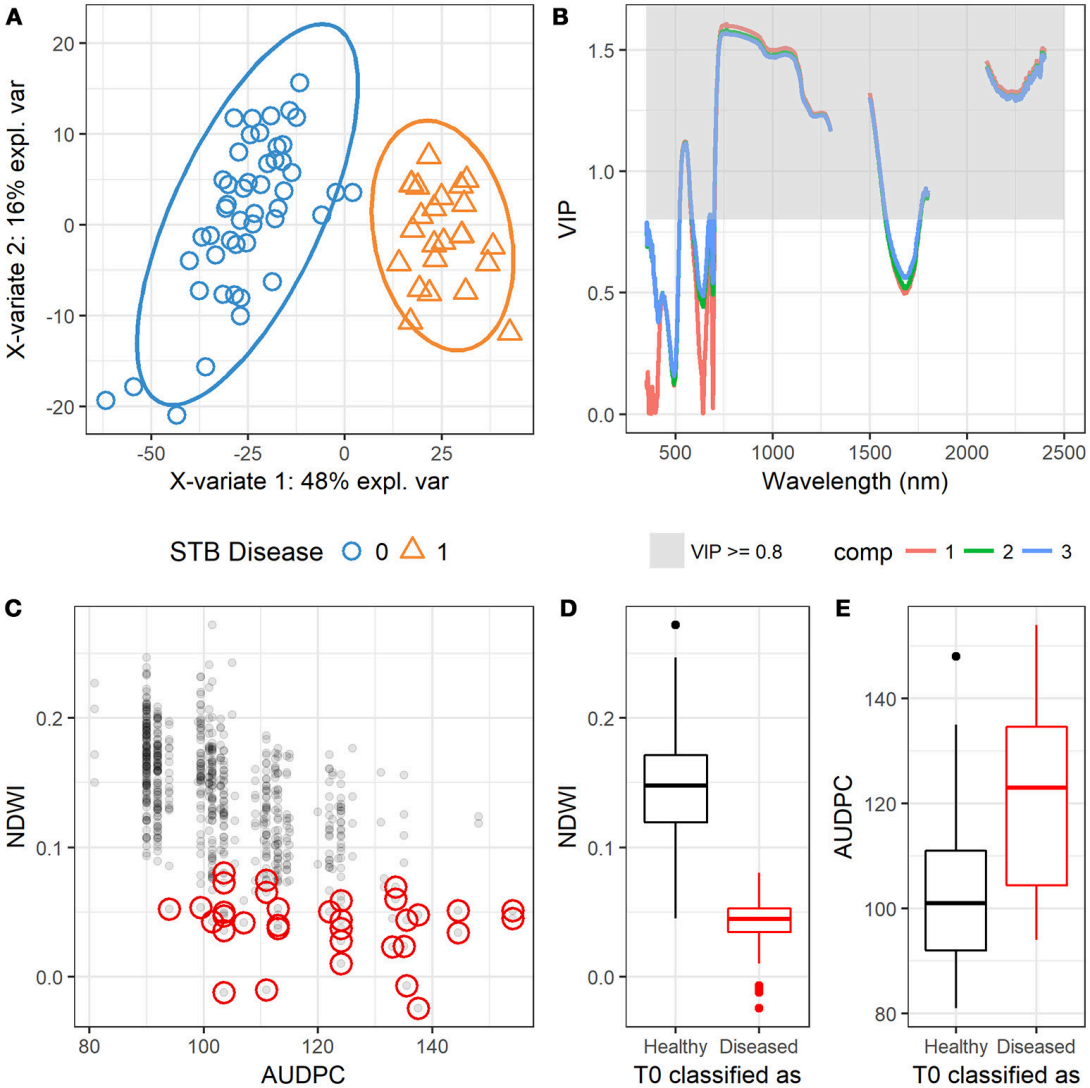
**(A)** Scatterplot shows the observations that are projected onto the first two components of the PLSDA model, suggesting the complete separation of the two groups (0 = healthy, 1 = diseased). **(B)** Variable importance in projections (VIP) scores at different wavelengths projected in the first three components (comp 1, 2, and 3). **(C)** Scatter plot shows the relationship between NDWI and AUDPC, and red circles highlight the observations of *Zymoseptoria tritici* un-treated plots (T0) that were classified as diseased canopies by the PLSDA model. **(D)** Boxplot shows the difference in NDWI between the correctly classified and misclassified T0 plots. **(E)** Boxplot shows the difference in AUDPC between the correctly classified and misclassified T0 plots.

Selection of highly informative bands could improve the efficiency and accuracy of PLSDA models for estimation, as some bands have low signal-to-noise ratio and some bands might be redundant in term of similar responses to certain characteristics of plants and canopy (Thenkabail et al., 2011). In this study, band selection for the PLSDA model was performed based on the VIP statistics (Wold et al., 2001), and a total of 1,086 spectral bands that yielded VIP scores higher than 0.8 were selected for the PLSDA model. Results showed that the PLSDA model allowed for complete separation between the diseased and healthy canopies (Figure 9A), and spectral bands at wavelength 520–580, 700–1,300, 1,500–1,580, 1,760–1,800 nm and 2100–2400 were effective for STB disease classification (Figure 9B, Table S1). Other studies have also shown that NIR and SWIR spectral bands are very efficient for the detection of plant diseases (Delalieux et al., 2009; Bauriegel and Herppich, 2014).

The trained PLSDA model was applied to the independent dataset [710 healthy (T0) and 48 diseased (T1) plots/canopies] to test the capability of classification. Results showed that the overall classification accuracy was 93% (Table 5). Model test sensitivity (i.e., TPR) was 95.8%, and test specificity (i.e., TNR) was 92.7%, i.e., only two diseased (T1) plots were misclassified as healthy plots (Table 5). 52 plots were classified as belonging to the diseased canopies, accounting for only 7% of the 710 healthy plots. This finding led us to consider whether the misclassified plots were planted to cultivars that were highly susceptible to STB. PLACL of individual leaves in the 52 plots ranged from 21 to 82 with a mean value of 50 (data not shown), placing these plots mainly in the upper range of the overall STB rating score (≥ 3, (Figure S7). In 22 of these plots, PLACL was higher than 50, indicating that these plots had significant STB infections despite the intensive use of fungicides. Hence, the classification of these plots as diseased by the model was reasonable (an expected misclassification), providing additional evidence for the power of this method. Moreover, the NDVI and NDWI values for these plots were relatively low compared to the correctly classified healthy plots (Figure 9C and Figure S7), in agreement with the negative correlations between NDVI or NDWI, on the one hand, and STB scores and AUDPC, on the other hand (Figure S1 and Figure 3). Incorrect classification of the 30 plots with PLACL values lower than 50 might be due to special phenotypic characteristics—for instance, a group of wheat genotypes having lighter green leaves. Further investigations will be needed to determine the cause of these misclassifications.

Calculated based on three independent measures of disease over time, AUDPC is often used in resistance breeding because it reduces the rating bias inherent in individual assessments (Li et al., 2006; Karisto et al., 2018). Here, the AUDPC values for the 52 misclassified plots spanned a very wide range compared to NDWI, which is in line with the capabilities of separation between diseased and healthy canopies (Figures 9D,E). NDVI indicates overall greenness of the canopy, and dark green canopies (high NDVI) are more likely to be visually rated at low STB scores. Results showed that there was a certain degree of consistency between the visual assessment and spectral indices using the visible region (e.g., NDVI, Figure S7), which also suggests that visual assessment might underestimate the infection when mild infection (e.g., scores ranged from 0 to 5 in this study) occurs in canopies of high NDVI. In contrast, NDWI improved separation as compared to NDVI and AUDPC, implying the possibility of hyperspectral canopy sensing of mild infections or early occurrence of STB infections using NIR to SWIR bands (Figure 9C). However, NDVI is also indicative of disease severity when the infection is severe (Jansen et al., 2014). Compared to visual assessments that depend largely on individual skill and training, canopy spectral metrics tend to be more reproducible and less prone to human bias (Steddom et al., 2005).

The PLSDA training dataset contains only four varieties, which might have constrained the power of the PLSDA model. However, the validation on an independent dataset of 331 varieties shows the promise of combining canopy hyperspectral measurements and PLSDA modeling for crop disease detection. Compared to spectral indices, the PLSDA model allowed for the best-balanced ability, yielding model test sensitivity and specificity of 95.8 and 92.7%, respectively and the overall accuracy of 93% (kappa = 0.60) (Table 5). Having the highest sensitivity, the PLSDA model suggests the great potential of sensing early infections before significant visible symptoms develop in the canopy.

Despite the promise of hyperspectral remote sensing for plant disease phenotyping, further investigations including a wider spectrum of plant diseases, an even larger number of crop varieties, and a broader range of environments will be required to improve crop disease detection and differentiation, and determine whether the desired precision, reproducibility and throughput needed for resistance breeding can be achieved (Furbank and Tester, 2011; O'Driscoll et al., 2014; Mahlein, 2016). Moreover, further investigations will be needed to determine whether canopy reflectance and discriminant analysis methods can differentiate STB from other symptoms such as nutrient or water stresses (Bürling et al., 2011). Future work should also investigate the possibility that fungicide applications might affect the comparison of canopy hyperspectral responses. Progress in addressing these questions will advance plant disease phenotyping and enable improved mapping for precise applications of fungicides in the field, especially from low-altitude sensor carrying platforms such as UAVs (Garcia-Ruiz et al., 2013; Yuan et al., 2016; Yang et al., 2017).

## Conclusions

In this study we investigated the feasibility of using canopy hyperspectral data to detect wheat STB infection in a population of 335 elite European wheat varieties. Our results demonstrated that hyperspectral indices used for the estimation of leaf chlorophyll and water status were negatively related to the canopy and leaf STB metrics, whereas the senescence related spectral indices were positively related. NDWI is more indicative of early infections compared to NDVI. These findings lead us to hypothesize that STB infections induced leaf biochemical changes and accelerated plant senescence, resulting in early changes in the spectral signature associated with water absorption. Full-spectrum analysis through the partial least squares (PLS) method allowed for the prediction of canopy- and leaf-level STB severity metrics, suggesting the promise of using canopy spectra as new tools to quantify STB damage across a wide array of wheat genotypes. PLS discriminant analysis empowered the canopy hyperspectral measurements to discriminate STB diseased canopies from healthy canopies with high accuracy, and showed the potential of using the NIR to SWIR region to discriminate diseased canopies in this study. Additionally, hyperspectral narrow bands in the red edge, NIR to SWIR regions were identified as highly efficient bands for disease discrimination. Our findings suggest that canopy hyperspectral measurements have great potential to facilitate high throughput plant phenotyping for disease resistance breeding. Further investigations that lead to a better understanding of the interaction between disease progress, canopy structure, and physiological senescence will further improve our ability to identify and measure diseases using remote sensing technologies.

## Author contributions

KY, AM, and AH designed the experiment. KY and JA performed the canopy spectral measurement. AM and PK performed the leaf sampling and leaf STB imagery assessment. FM performed the visual assessment of STB infection. KY analyzed the data and wrote the draft. BM, AW, and AH supervised the project and designed the research. All authors read, revised, and approved the final manuscript.

### Conflict of interest statement

The authors declare that the research was conducted in the absence of any commercial or financial relationships that could be construed as a potential conflict of interest.
