# Supplementary material for: Hyperspectral Canopy Sensing of Wheat Septoria Tritici Blotch Disease
Source: Front Plant Sci. 2018 Aug 17;9:1195. doi: 10.3389/fpls.2018.01195 (PMC6108383; doi:10.3389/fpls.2018.01195)
Supplement: Supplementary file 1 [file Data_Sheet_1.docx]

# Supplementary Material


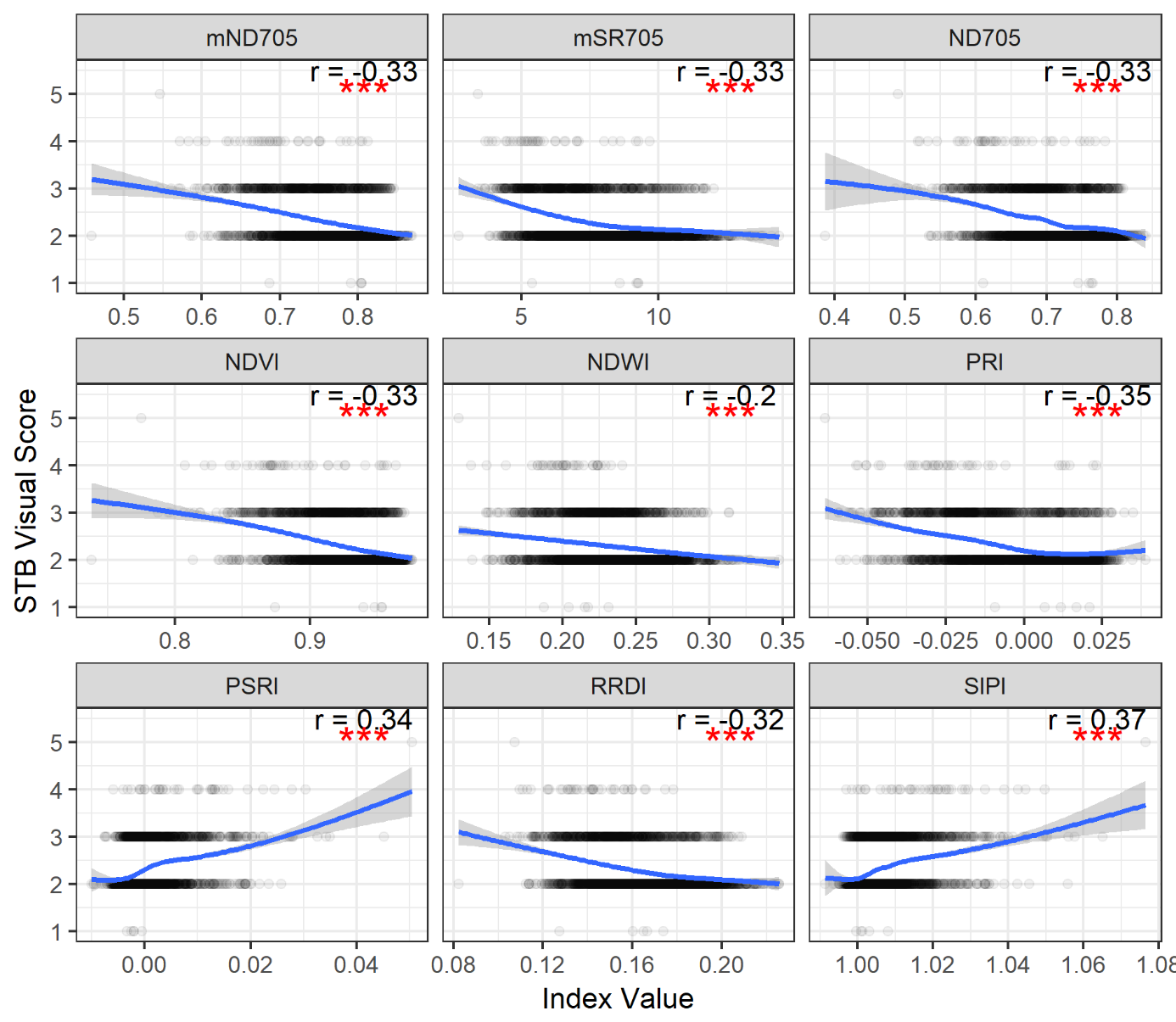


Figure S1. STB visual scores plotted as a function of spectral indices. Correlation coefficients (r) are for the Spearman’s correlations between the spectral indices and STB visual scores across three assessments (*** p < .001). Loess fit lines are added to the scatter plot to show the trend of the relationship. Results are based on the data of the whole population (335 varieties) used in the main experiment.


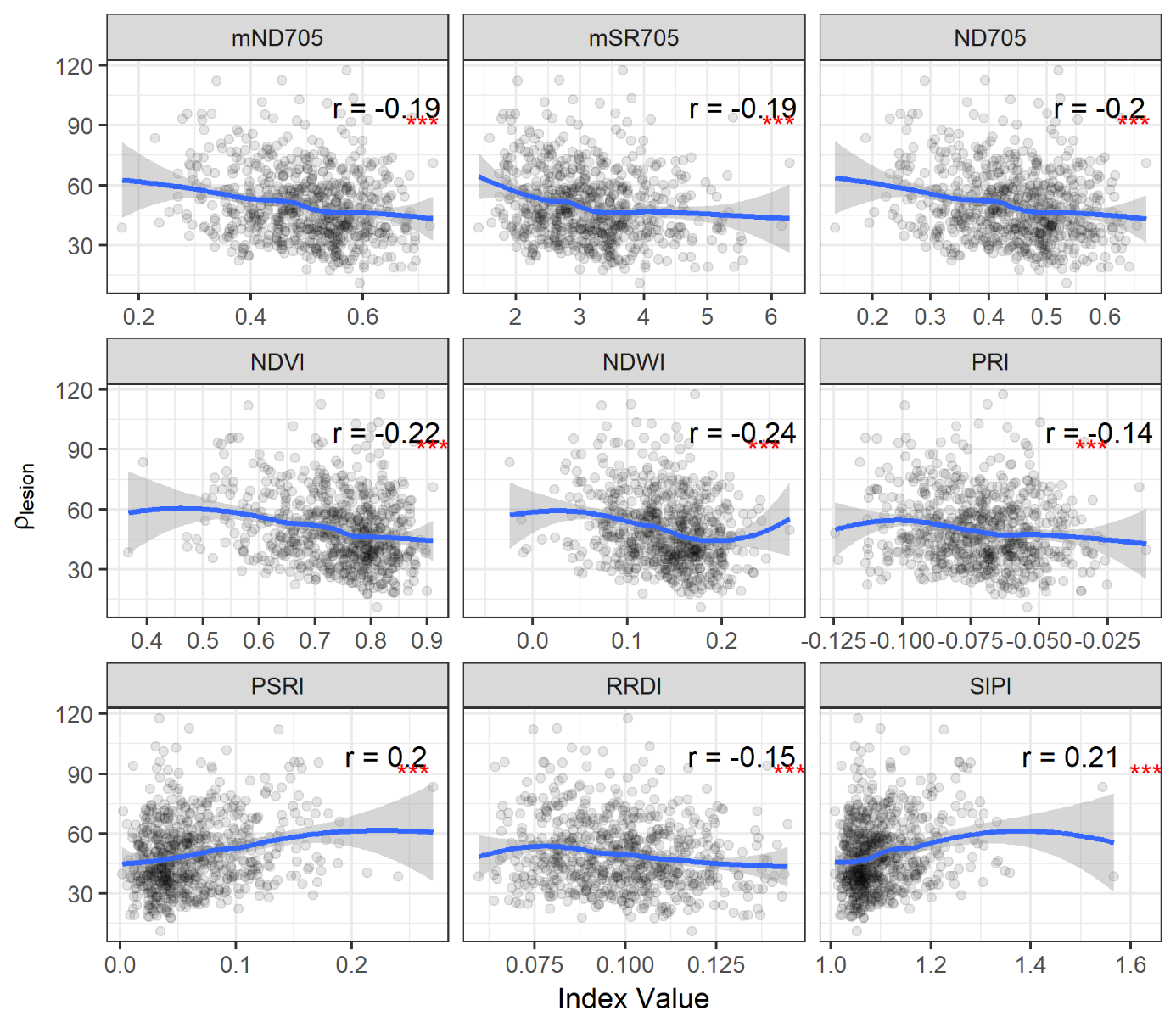


Figure S2. STB density of pycnidia per unit lesion area (ρ_lesion_) plotted as a function of spectral indices for the second collection. Correlation coefficients (r) are for the Spearman’s correlations between the spectral indices and ρ_lesion_ (*** p < .001). Loess fit lines are added to the scatter plot to show the trend of the relationship. Results are based on the data of the whole population (335 varieties) used in the main experiment.


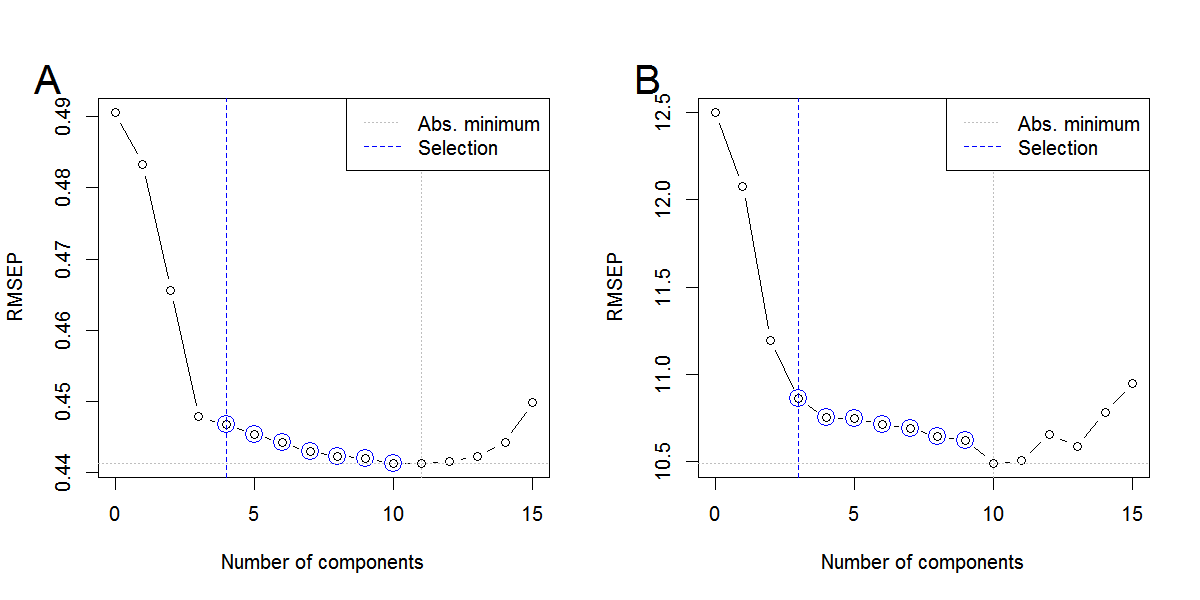


Figure S3. Number of components used for the PLSR models for (A) STB scores and (B) AUDPC. A permutation approach was employed to test whether adding a new component significantly decreases the RMSEP.


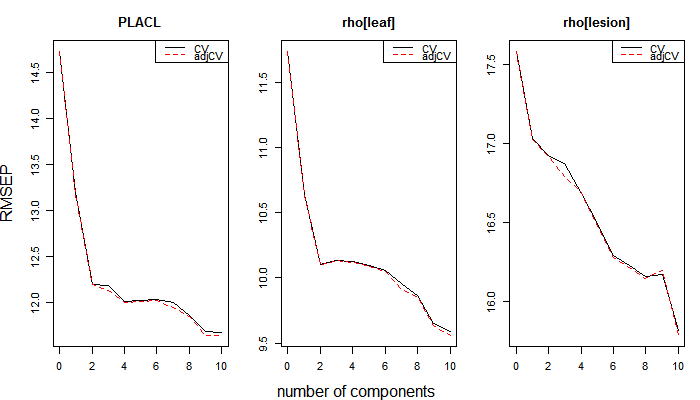


Figure S4. Root mean square errors of prediction (RMSEP) when using different number of components for the PLSR model for PLACL, ρ-leaf and ρ-lesion. It can be observed that 4-6 components allowed for significant reduction of prediction errors.


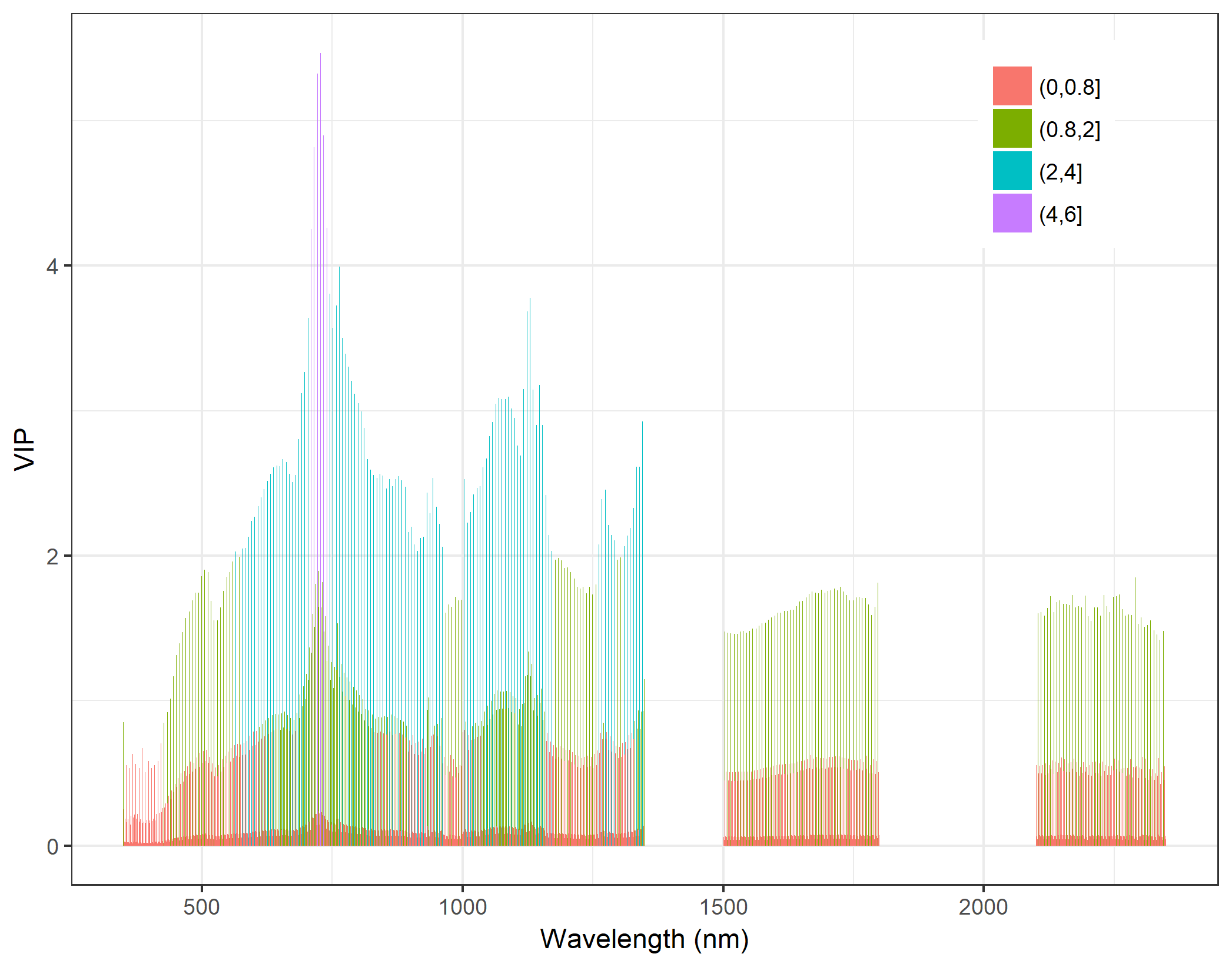


Figure S5. Variable importance in projections (VIP) scores of the PLSR model using the three leaf STB metrics as response variables. VIP scores shown here are calculated with 6 components.


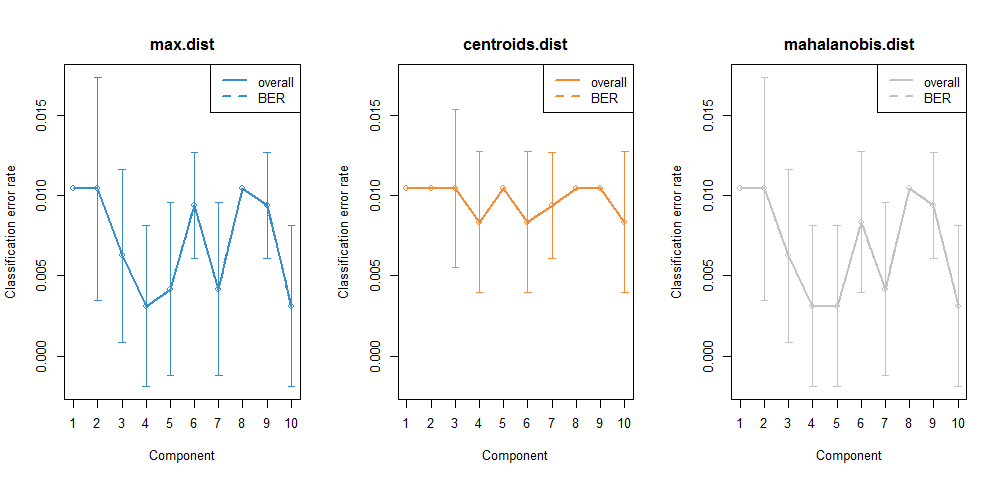


Figure S6. Classification error rate for the PLSDA model when using different numbers of components. Results suggested that the use of 4 components allowed for significant reduction of prediction errors for the PLSDA model.


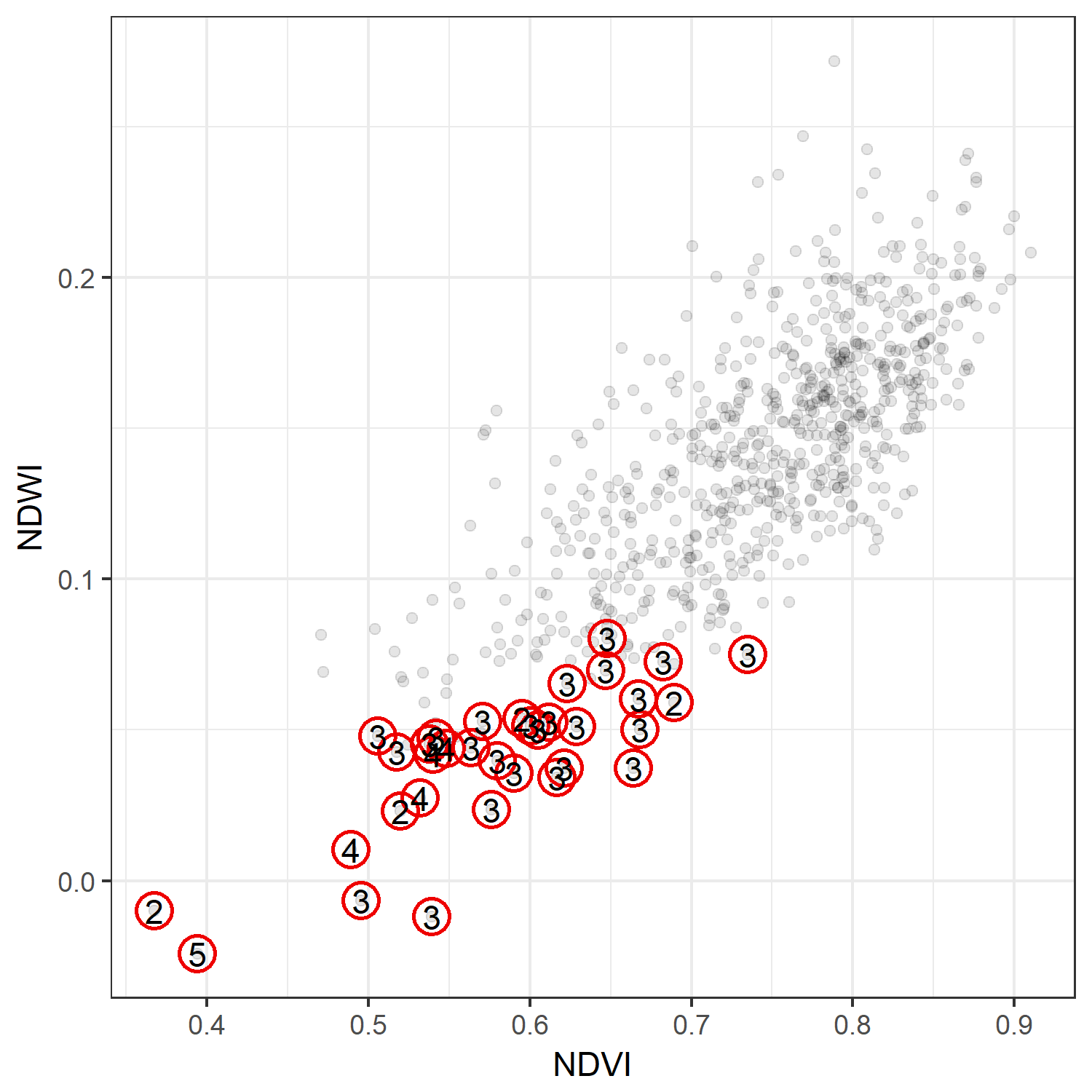


Figure S7. Scatter plots show the relationship between NDW and NDVI, and red circles highlight the healthy plots that were classified as diseased plots by the PLSDA model. Numbers in the red circles are the visual rating scores of corresponding plots.
